# Acute Cervical Dystonia Induced by Clebopride

**DOI:** 10.1155/2017/2834349

**Published:** 2017-11-28

**Authors:** Jin Kyo Choi, Jin Yong Hong

**Affiliations:** ^1^Pohang City Namgu Health Center, Pohang, Republic of Korea; ^2^Department of Neurology, Yonsei University Wonju College of Medicine, Wonju, Republic of Korea

## Abstract

Antidopaminergic drugs are known to induce extrapyramidal symptoms. Clebopride, a dopamine antagonist, also can produce parkinsonism, tardive dyskinesia, tardive dystonia, hemifacial dystonia, or oculogyric crisis; however, acute dystonic reaction caused by clebopride has not been reported in adults. We report two young men who experienced acute cervical dystonia within a few days of taking clebopride. The patients recovered after discontinuation of the drug. Physicians prescribing clebopride should be aware of the adverse effects of this drug.

## 1. Introduction

Extrapyramidal symptoms can be induced by many agents. Antiemetics are widely used to treat patients with gastrointestinal problems; however, their antidopaminergic effect makes them the most common cause of drug-induced movement disorders [1]. Clebopride, a nonselective benzamide, also has high affinity for D2, D3, and D4 receptors [2]; thus, it acts as a dopamine receptor-blocking agent. Although parkinsonism [3, 4], tardive dyskinesia [4], tardive dystonia [5, 6], hemifacial dystonia [7], and oculogyric crisis [8] have been reported to be caused by clebopride, acute cervical dystonia has not been observed in adults treated with this drug. Here, we present two young men who presented with acute cervical dystonia related to the administration of clebopride.

## 2. Case Presentation

### 2.1. Case  1

A 26-year-old man visited our hospital for stiffness and pain in the neck that had persisted for one day. He felt spasms and twisting of the neck the day before visiting our hospital and reported that intravenous injection of diazepam in another hospital was ineffective. A physical examination showed increased tone in all muscles of the neck and a dystonic posture with torticollis to the left side (video). However, no neurological abnormality including dysarthria, motor weakness, sensory change, ataxia, bradykinesia, or tremor was found. He had taken clebopride (680 *μ*g, three times a day), simethicone (200 mg, three times a day), tiropramide (100 mg, three times a day), ranitidine (150 mg, three times a day), almagate (500 mg, three times a day), and pantoprazole (10 mg, once a day) for reflex esophagitis for 7 days before developing dystonia. There was no other drug before treatment for reflex esophagitis. Brain magnetic resonance imaging (MRI) results were normal and there was no mutation in either DYT1 or PANK2 genes. Results of other laboratory tests including those for serum copper, total urine copper during 24 hours, serum ceruloplasmin, and serum ferritin were also within normal ranges.

The patient discontinued taking the drugs on the visit day, and then we prescribed clonazepam (0.25 mg, twice a day), eperisone (50 mg, twice a day), and benztropine (1 mg, twice a day) for symptom relief. Dystonia remitted completely 7 days after the change in medications and did not recur after the symptomatic treatment was stopped.

### 2.2. Case  2

An 18-year-old man visited our emergency department for neck pain and spasm. The symptom developed suddenly 2 hours before the visit. Physical examination showed increased muscle tone and dystonic posture of the neck, but there was no neurological abnormality such as bradykinesia, ataxia, motor weakness, or hypoesthesia. He had taken clebopride (680 *μ*g, three times a day), simethicone (200 mg, three times a day), and tiropramide (100 mg, three times a day) for nausea and vomiting for 2 days before the appearance of dystonia. He had neither medicosurgical history nor oral medication before the gastric symptom. Results of brain MRI and routine serologic study were normal.

The patient stopped clebopride and was started on baclofen (5 mg, three times a day). Dystonic symptoms disappeared within 5 days and did not recur after discontinuation of baclofen.

## 3. Discussion

To our knowledge, this is the first report of acute cervical dystonia induced by clebopride in adults. Clebopride has been reported to cause acute events as hypertonic reaction in children [9] and oculogyric crisis in young adults [8], but only one case of acute hemifacial dystonia was observed in adults [7]. Other extrapyramidal symptoms that developed in adults were chronic reactions including parkinsonism [3, 4] or tardive dyskinesia [4].

In our patients, cervical dystonia developed within a few days of clebopride use. Therefore, it can be classified as an acute dystonic reaction (ADR). About 50% of the cases of ADR occur within 48 hours and 90% within 5 days of drug treatment [10]. ADR is induced mainly by antipsychotics [11], but diverse drugs including fluoxetine [12], domperidone [13], and metoclopramide [14, 15] can cause it. Young age, higher potency and dose of the offending drug, and psychiatric illness are known risk factors for ADR [15]. Similar to children and young adults showing ADR in the previous report [8, 9, 15], our patients were also young. Therefore, ADR may be related to maturity or activity of the dopaminergic system, although its mechanism is currently unclear.

The two cases reported here suggest that acute cervical dystonia should be included in the spectrum of extrapyramidal adverse events of clebopride administration, and physicians who prescribe this drug should be aware of it.

## Supplementary Materials

Supplementary Materials Supplementary Video. The patient shows increased muscle tone in the whole neck and torticollis to the left side.
